# Chirality, a new key for the definition of the connection and curvature of a Lie-Kac superalgebra

**DOI:** 10.1007/jhep01(2021)111

**Published:** 2021-01-20

**Authors:** Jean Thierry-Mieg

**Affiliations:** NCBI, National Library of Medicine, National Institute of Health, 8600 Rockville Pike, Bethesda, MD 20894, U.S.A.

**Keywords:** Differential and Algebraic Geometry, Non-Commutative Geometry, Chern-Simons Theories

## Abstract

A natural generalization of a Lie algebra connection, or Yang-Mills field, to the case of a Lie-Kac superalgebra, for example SU(m/n), just in terms of ordinary complex functions and differentials, is proposed. Using the chirality χ which defines the supertrace of the superalgebra: STr(…)=Tr(χ…), we construct a covariant differential: D=χ(d+A)+Φ, where A is the standard even Lie-subalgebra connection 1-form and Φ a scalar field valued in the odd module. Despite the fact that Φ is a scalar, Φ anticomtes with (χA) because χ anticommutes with the odd generators hidden in Φ. Hence the curvature *F* = *DD* is a superalgebra-valued linear map which respects the Bianchi identity and correctly defines a chiral parallel transport compatible with a generic Lie superalgebra structure.

## 1 Introduction

In differential geometry and in Yang-Mills theory, the Lie algebra-valued connection *A* and the curvature 2-form *F* = *dA* + *AA* are fundamental concepts. A natural generalization to the case of a (simple) Lie-Kac superalgebra [1], for example SU(*m/n*), raises a sign issue. The difficulty stems from the fact that 1-forms naturally anticommute, so the product *AA* appearing in the definition of the curvature *F* hides a matrix commutator $AA=\begin{array}{c} \frac{1}{2}A^{a}A^{b}\left[\lambda_{a},\lambda_{b}\right] \end{array}$ which, as desired, closes in a Lie algebra. But in a superalgebra, we need to associate to the odd generators a commuting field Φ such that the product ΦΦ is symmetric and hides an anticommutator $\text{ΦΦ}=\frac{1}{2}{\text{Φ}}^{i}{\text{Φ}}^{j}\left\{\lambda_{i},\lambda_{j}\right\}$. Yet this commuting scalar field Φ must anticommute with *A* in order to generate, in the even-odd sector, a matrix commutator [*λ*_*a*_, *λ*_*i*_], and Φ must be odd as an element of the differential calculus so that the curvature 2-form *F* = *DD* remains a linear map.

Several constructions have been proposed. They have in common that they postulate that the scalar fields Φ, called *L* in Quillen [2, 3], are odd relative to the Yang-Mills 1-forms *A*. The *L* are often represented as 1-forms relative to an enlarged space which can be a super-space involving anticommuting Grassman coordinates [4–6], or a discrete space, for example *Z*_2_, equipped with discrete differentials [7–14]. But these odd *L* fields cannot easily be represented in quantum field theory, where bosons necessarily commute. Therefore *L* cannot correspond to the Higgs fields of the standard model of the fundamental interactions.

In this note, using a slight modification of the usual formalism of covariant differentials and connections, we construct a surprisingly simple universal solution to this problem. Any superalgebra [1] carries a *Z*_2_ chirality operator χ which commutes with the even generators spanning the Lie subalgebra, anticommutes with the odd generators spanning the odd module, and defines the supertrace STr(…)=Tr(χ…). Consider a standard 1-form *A*, valued in the even Lie subalgebra, together with a scalar Φ valued in the odd module, and let us define the new chiral covariant differential $\tilde{D}=\chi (d+A)+\text{Φ}$. This algebraic structure implies three direct consequences. The curvature $\tilde{F}={\tilde{D}}^{2}$ is valued in the adjoint representation of the superalgebra. It defines a linear map. It satisfies the Bianchi identity $\tilde{D}\tilde{F}=0$. The proof relies on 3 rules: χ*A* anticommutes with χ*A* since *A* is a 1-form; Φ commutes with Φ since Φ is a scalar; finally χ*A* anticommutes with Φ, despite the fact that Φ^*i*^ is a standard commuting complex-valued function, because χ anticommutes with the odd generators *λ*_*i*_ hidden in $\text{Φ}={\text{Φ}}^{i}\lambda_{i}$.

As a result, $\tilde{D}$ defines a parallel transport on the super-principal bundle, where the base space is an ordinary differential manifold and the fiber is isomorphic to a Lie-Kac superalgebra. $\tilde{D}$ is defined in terms of standard complex-valued functions, without using non standard Grassman coordinates or discrete differentials. This natural construction may open the way to a reanalysis of the Ne’eman-Fairlie SU(2/1) superalgebraic model of the electroweak chiral interactions of leptons [15, 16] and quarks [17, 18]

## 2 Definition of a superalgebra

Let us consider a finite dimensional basic classical Lie-Kac superalgebra [1]. The superalgebra acts on a *Z*_2_ graded finite dimensional vector space $V=V_{0}+V_{1}$ over the complex numbers. The chirality matrix χ is diagonal, with eigenvalue 1 on the *V*_0_ and −1 on *V*_1_. χ defines the supertrace

(2.1) STr(…)=Tr(χ…).

Each generator is represented by a finite dimensional matrix of complex numbers. Note that in our approach, we do not need anticommuting Grassman numbers. The even generators are denoted *λ*_*a*_ and the odd generators *λ*_*i*_. χ commutes with the *λ*_*a*_ and anticommutes with the *λ*_*i*_

(2.2) $$\left[\chi ,\lambda_{a}\right]=\left\{\chi ,\lambda_{i}\right\}=0.$$

The *λ* matrices close under (anti)-commutation

(2.3) $$\left[\lambda_{a},\lambda_{b}\right]=f_{ab}^{c}\lambda_{c},\;\left[\lambda_{a},\lambda_{i}\right]=f_{ai}^{j}\lambda_{j},\;\left\{\lambda_{i},\lambda_{j}\right\}=d_{ij}^{a}\lambda_{a},$$

and satisfy the super-Jacobi relation with 3 cyclic permuted terms:

(2.4) $${\left(-1\right)}^{AC}\text{\{}\lambda_{A},\text{\{}\lambda_{B},\lambda_{C}\text{]]}+{\left(-1\right)}^{BA}\text{\{}\lambda_{B},\text{\{}\lambda_{C},\lambda_{A}\text{]]}+{\left(-1\right)}^{CB}\text{\{}\lambda_{C},\text{\{}\lambda_{A},\lambda_{B}\text{]]}=0.$$

where the mixed bracket denotes either a commutator or an anticommutator as needed. The quadratic Casimir tensor (*g*_*ab*_, *g*_*ij*_), also called the super-Killing metric, is defined as

(2.5) $$\begin{array}{l} g_{ab}=\frac{1}{2}STr\left(\lambda_{a}\lambda_{b}\right),\\ g_{ij}=\frac{1}{2}STr\left(\lambda_{i}\lambda_{j}\right) \end{array}$$

The even part *g*_*ab*_ of the metric is as usual symmetric, but because the odd generators anticommute with the chirality hidden in the supertrace (2.1), its odd part *g*_*ij*_ is antisymmetric: $Tr\left(\chi \lambda_{j}\lambda_{i}\right)=-Tr\left(\lambda_{j}\chi \lambda_{i}\right)=-Tr\left(\chi \lambda_{i}\lambda_{j}\right)$. The structure constants can be recovered from the supertrace of a product of 3 matrices

(2.6) $$\begin{array}{c} f_{abc}=g_{ae}f_{bc}^{e}=\frac{1}{2}STr\left(\lambda_{a}\left[\lambda_{b},\lambda_{c}\right]\right),\\ f_{iaj}=g_{ik}f_{aj}^{k}=\frac{1}{2}STr\left(\lambda_{i}\left[\lambda_{a},\lambda_{j}\right]\right),\\ d_{aij}=g_{ae}d_{ij}^{e}=\frac{1}{2}STr\left(\lambda_{a}\left\{\lambda_{i},\lambda_{j}\right\}\right). \end{array}$$

In an abstract way, a Lie-Kac superalgebra is specified by providing a set of even and odd generators Λ^*a*^ and Λ^*i*^ satisfying closure and the graded Jacobi identity, plus a chirality operator *X* commuting/anticommuting with the even/odd generators. A finite dimensional linear representation ρ over the complex numbers is defined by providing a set of finite dimensional complex matrices denoted *λ*^*a*^ and *λ*^*i*^, such that ρ(Λ)=λ, and a grading matrix χ=ρ(X) satisfying the same relations. The matrix χ is not an addition, but an intrinsic part of the definition of the linear representation ρ. It will play a central role in our new definition of a superalgebra superconnection.

## 3 Lie algebra connections respect the Bianchi identity

In the Lie algebra case, a connection *A* is a Lie algebra-valued 1-form

(3.1) $$A=A_{\mu }^{a}(x)dx^{\mu }\lambda_{a},$$

where $A_{\mu }^{a}(x)$, the Yang-Mills vector-field of the physicists, is an ordinary commuting complex valued function, and *dx*^*μ*^, the exterior differentials of the coordinates, are anticommuting 1-forms. *A* can be combined with the exterior differential $d=\partial_{\mu }dx^{\mu }$ to construct a covariant differential

(3.2) D=d+A.

Let us iterate the action of *D* on a vector *ψ*. Since *d* satisfies the graded Leibniz rule

(3.3) d(A…)=(dA)…−Ad(…)

we find that *D*^2^ is tensorial, i.e. defines a linear map with respect to scalar functions, as $D^{2}\psi$ is independent of dψ

(3.4) $$D^{2}\psi =(d+A)(d+A)\psi =(dA+AA)\psi +0d\psi$$

allowing us to define the curvature 2-form

(3.5) $$F=D^{2}=dA+AA$$

The second crucial observation is that, because the *A* are 1-forms, the product *AA* is antisymmetric in (*a, b*) and hence proportional to the commutator of the *λ* matrices

(3.6) $$AA=\frac{1}{2}A_{\mu }^{a}A_{\nu }^{b}dx^{\mu }dx^{\nu }\left[\lambda_{a},\lambda_{b}\right]$$

and since the commutator closes in the algebra (2.3), the curvature 2-form F is valued in the adjoint representation of the Lie algebra

(3.7) $$F=F^{a}\lambda_{a}=\left(dA^{a}+\frac{1}{2}f_{bc}^{a}A^{b}A^{c}\right)\lambda_{a}.$$

Let us now verify that the triple action of *D* is associative:

(3.8) D(DD)ψ=(DD)Dψ.

This condition is equivalent to the Bianchi identity

(3.9) DF=dF+AF−FA=0.

In this equation, the terms linear in *dA*, coming from F=dA+AA, cancel out and we are left with the constraint

(3.10) $$A^{b}A^{c}A^{d}f_{be}^{a}f_{cd}^{e}=0.$$

Because the *A* 1-forms anticommute, this constraint (3.10) is satisfied thanks to the Jacobi identity (2.4). Thus, for a Lie algebra-valued connection 1-form, the Bianchi identity (3.9) is satisfied and the covariant differential *D* is associative (3.8).

## 4 About superconnections

Let us now try to extend those definitions to a Lie-Kac superalgebra. In his seminal paper on superconnections, Quillen [2, 3] explains that if we define the covariant differential as

(4.1) $$\tilde{D}=\tilde{d}+\tilde{A}$$

then the connection form $\tilde{A}$ must be odd with respect to the differential calculus, meaning that ${\tilde{d}}^{2}$ must vanish and $\tilde{d}$ must satisfy the graded Leibniz rule (3.3). As shown in the previous section, these rules are naturally valid for the exterior differential *d* and the 1-forms *A*, if *A* is a Lie algebra valued connection. The question is to find a generalization if $\tilde{A}$ is valued in the adjoint representation of a Lie-Kac superalgebra.

A first naive guess would be to construct the superconnection as a 1-form

(4.2) $$D=d+A=dx^{\mu }\left(\partial_{\mu }+A_{\mu }^{a}\lambda_{a}+A_{\mu }^{i}\lambda_{i}\right).$$

However, this definition would be inconsistent because the curvature *F* would involve the commutator of the odd matrices

(4.3) $$F=dA+AA=\ldots +A_{\mu }^{i}A_{\nu }^{j}dx^{\mu }dx^{\nu }\frac{1}{2}\left[\lambda_{i},\lambda_{j}\right]$$

which does not close on the even matrices.

The next possibility is to define the theory over superspace [4–6]. This method introduces a lot of subtleties. As usual in supersymmetric theories, each component field *A*(*x,θ*) can be developed as a finite polynomial over the Grassman coordinates *θ*, implicitly generating a great number of auxiliary fields. But the exterior differentials *dθ* of the Grassman coordinates generate an even larger complexity. The *dθ* commute, so polynomials of arbitrary degree in *dθ* can exist. But in a way, this approach cannot solve our original question. Indeed, the intrinsic geometry of Elie Cartan, which deals with exterior differential and exterior forms, is by construction independent of the choice of coordinates on the base space, which can be a standard differential manifold (*x*), or a superspace (*x,θ*). For example, the Lie algebra covariant differential over superspace simply reads D=d+A(x,θ) where $d=\partial_{\mu }dx^{\mu }+\partial_{\theta }d\theta$. It is therefore apparent that the introduction of a superspace does not resolve the paradoxical sign rules that we described in the introduction, unless we restrict the even components of the connection *A*^*a*^ to depend only on *dx* and the odd components *A*^*i*^ only on $d\theta :A=A_{\mu }^{a}\lambda_{a}dx^{\mu }+A^{i}\lambda_{i}d\theta$. But such an assumption contradicts the essence of Cartan’s intrinsic calculus since this constraint is not invariant under a generic super-rotation of the coordinates.

In 1982, with Ne’eman [19], we introduced a superconnection as the odd part of the de Rham complex of forms of all degrees, valued in a Lie superalgebra.

(4.4) $$\tilde{A}=\text{Φ}+A+B+\ldots ={\text{Φ}}^{i}\lambda_{i}+A^{a}\lambda_{a}+B^{i}\lambda_{i}+\ldots$$

where Φ is a scalar field valued in the odd module of the superalgebra, *A* is a 1-form valued in the even subalgebra, *B* a 2-form valued in the odd module and so on. In this method, when we compute ${\left(d+\text{Φ}+A\right)}^{2}$, the term in *AA* gives as usual the commutator of the even generators (3.6) and since Φ is a scalar, the term in ΦΦ is symmetric and generates as desired the anticommutator of the odd generators

(4.5) $$F=d(A+\text{Φ})+(A+\text{Φ})(A+\text{Φ})=\ldots +{\text{Φ}}^{i}{\text{Φ}}^{j}\frac{1}{2}\left\{\lambda_{i},\lambda_{j}\right\}$$

However, d(Φ…) does not obey the graded Leibniz rule (3.3) but gives a plus sign which breaks the tensorial nature of the curvature *F*

(4.6) DDψ=…+d(Φψ)+Φdψ=…+(dΦ)ψ+2Φdψ

which now depends on dψ. At the same time, this proposition does not generate in the even-odd sector the desired commutator [*λ*_*a*_, *λ*_*i*_], but an anticommutator

(4.7) $$A\text{Φ}+\text{Φ}A=A^{a}{\text{Φ}}^{i}\left(\lambda_{a}\lambda_{i}+\lambda_{i}\lambda_{a}\right)$$

which does not close in the superalgebra. The same problem affects the product *AB* and all other terms in the even-odd sector.

In 1985, Quillen [2, 3] proposed a general construction applicable to non-trivial bundles and resolved this problem by adding to the Yang-Mills covariant differential D=d+A a new *L* term

(4.8) D=d+A+L

where *L* is valued in the odd module of the superalgebra $L=L^{i}\lambda_{i}$ and $L^{i}$ is by definition odd with respect to the differential calculus, meaning that *L* anticommutes with *A* and satisfies the graded Leibniz rule. In essence, Quillen postulates that *L* takes values in another algebra which anticommutes with the exterior differentials. This method was later adopted by Ne’eman and Sternberg who postulate that $\omega_{0}L_{01}=-L_{01}\omega_{0}$, see equation 1.3ab of [20] or section 5.8 of [21]. This is a correct mathematical construction, but it is not applicable to physics. The limitation resides in its transposition to quantum field theory (QFT). The connection 1-forms $A=A_{\mu }^{a}\lambda_{a}dx^{\mu }$ are naturally represented by the Yang-Mills vector bosons $A_{\mu }^{a}$, the even connection $L=L^{i}\lambda_{i}$ looks like a scalar fields *L*^*i*^ which, in the SU(2/1) case, has the quantum number of a Higgs Boson. Unfortunately bosons commute. Therefore there is no clear way to represent in QFT the required anticommutativity of $A_{\mu }^{a}$ and *L*^*i*^. This is probably why, up to now, the literature on the superalgebraic SU(2/1) model of the weak interactions [9, 10, 12–20] does not go beyond a classical analysis and never mentions renormalization and the Feynman diagrams of QFT.

## 5 New definition of a chiral superconnection satisfying the Bianchi identity

Let us now introduce a very simple new definition which naturally applies to any Lie-Kac superalgebra. Consider the exterior differential

(5.1) $$\begin{array}{ll} \tilde{D}=\tilde{d}+\tilde{A}, & \\ \tilde{d}=\chi d, & \tilde{A}=\chi A+\text{Φ},\\ \tilde{D}=\chi dx^{\mu }\left(\partial_{\mu }+A_{\mu }^{a}\lambda_{a}\right)+{\text{Φ}}^{i}\lambda_{i}, & \end{array}$$

where χ is the chirality operator instrumental in the definition of the supertrace (2.1) and *D* is the standard Lie algebra covariant differential D=d+A (3.2). This definition is valid for any superalgebra, since they are all equipped with a chirality operator. It does not involve Grassman numbers. $A_{\mu }^{a}(x)$ and ${\text{Φ}}^{i}(x)$ are standard complex-valued fields, respecting the spin-statistics theorem. As in equation (3.6), the $\tilde{F}=\ldots +AA$ term generates the desired commutator of the even *λ*_*a*_ generators. As in equation (4.5), the $\tilde{F}=\ldots +\text{ΦΦ}$ term generates the desired anticommutator of the odd *λ*_*i*_ generators. The new result is that χd and χA, which are 1-forms, anticommute with the scalar field Φ, not because of their exterior degree, but because the odd matrices *λ*_*i*_, hidden in $\text{Φ}={\text{Φ}}^{i}\lambda_{i}$, anticommute (2.2) with the chirality operator χ which defines the supertrace (2.1). As a consequence, $\left(\tilde{d}=\chi d\right)$ satisfies the graded Leibniz rule (3.3)

(5.2) $$\tilde{d}(\tilde{A}\ldots )=(\tilde{d}\tilde{A})\ldots -\tilde{A}\tilde{d}(\ldots ),$$

the sign problem of equation (4.6) disappears and the curvature 2-form $\tilde{F}$ defines a linear map as $\tilde{D}\tilde{D}\text{Ψ}$ does not depend on dΨ. Moreover, since χA anticommutes with Φ, all the commutators and anticommutators have the desired signs and $\tilde{F}$ is valued in the adjoint representation of the superalgebra:

(5.3) $$\begin{array}{l} \tilde{D}\tilde{D}\psi =\tilde{F}\psi =(\overset{˘}{F}+\overset{˘}{G})\psi ,\\ \;\overset{˘}{F}=F+\text{ΦΦ},\;\overset{˘}{G}=\overset{˘}{D}\text{Φ}, \end{array}$$

where the covariant differential $\overset{˘}{D}\text{Φ}$ contains the usual Lie algebra even-odd commutator

(5.4) $$\overset{˘}{D}\text{Φ}=\chi d\text{Φ}+\chi A\text{Φ}+\text{Φ}\chi A=\chi (d\text{Φ}+A\text{Φ}-\text{Φ}A).$$

Please observe how rule (2.2) defining the gradation of a superalgebra implies that Φχ=−χΦ thus changing the wrong anticommutator of equation (4.7) into the desired commutator (5.4). The new covariant differential $\tilde{D}$ is associative

(5.5) $$\tilde{D}(\tilde{D}\tilde{D})\psi =(\tilde{D}\tilde{D})\tilde{D}\psi$$

because the Bianchi identity

(5.6) $$\tilde{D}\tilde{F}=\tilde{d}\tilde{F}+\tilde{A}\tilde{F}-\tilde{F}\tilde{A}=0$$

is satisfied thanks to the super-Jacobi identity (2.4) since all the terms trilinear in (A,Φ) appear with the proper combinations of signs.

It may seem that an alternative construction: $D=\chi \tilde{D}=d+A+\chi \text{Φ}$, closer to [2, 21], could also work, but this is not the case. Since *d* commutes with χ,χΦ is even relative to the differentials *d*. Thus, the term in χΦdψ does not cancel out and the curvature does not define a linear map. This problem subsists for any definition of the type $D=d+{\text{Φ}}^{i}\mu_{i}$ where ${\text{Φ}}^{i}$ is a commuting scalar and *μ*_*i*_ an arbitrary matrix of complex numbers. The cornerstone of our new consistent definition is to join the chirality to the exterior differential $\tilde{d}=\chi d$ so that Φ becomes odd relative to $\tilde{d}$. The definition of Quillen D=d+A+L is consistent because he assumes that *L* is valued in an additional graded algebra odd relative to *d*, but then *L* cannot be represented in quantum field theory by a commuting scalar Boson and cannot be interpreted as the Higgs field of the standard model. We circumvent this problem by defining Φ as a standard commuting complex function and decorating the differential by the chirality matrix $\tilde{d}=\chi d$. Following Occam, this definition is also more economic than Quillen’s because χ is not an additional operator but a constitutive element of the definition of a linear representation of a Lie-Kac superalgebra.

Please notice that in these equations ψ does not denote a space-time spinor but an element of a generic linear representation of the superalgebra. Similarly, χ acts on ψ and anticommutes with the odd generators of the superalgebra but is not necessarily related to the *γ*_5_ chirality operator of the spinor space. The actual pairing of χ and *γ*_5_ as a representation of the *CP* invariance of the weak interactions is discussed in [22].

## 6 Application to Quantum Field Theory

The distinction between the even and odd generators of a superalgebra is intrinsic, they commute or anticommute with the chirality operator defining the supertrace (2.2). However, the carrier space $V=V_{0}+V_{1}$ is intrinsically symmetric. Presenting it as a *Z*_2_ graded space is in a way misleading because the two parts stand on equal footing. It is split, but it does not carry the distinct nature of 0 and 1 under addition and multiplication. There is no good reason to associate *V*_0_ to bosons and *V*_1_ to fermions, or vice-versa. For example, the quadratic super-Casimir operator is a multiple of the identity with the same eigenvalue *k* on *V*_0_ and on *V*_1_. It is more natural to think of χ as the chirality and to think of a superalgebra as mapping left to right fermions and vice versa [9, 15, 16, 21], rather than bosons to fermions [23].

Consider now the Dirac equation associated to our new chiral superconnection,

(6.1) $$\left(\chi \left(\partial_{\mu }+A_{\mu }\right)\gamma^{\mu }+\text{Φ}\right)\psi =0.$$

The presence of the chirality operator in front of the space-time derivative *∂*_*μ*_ solves the important problem of the sign of the energy of the vector Boson $A_{\mu }^{a}$. In a superalgebra, the natural normalization of the even matrices involves the supertrace

(6.2) $$STr\left(\lambda_{a}\lambda_{b}\right)=Tr\left(\chi \lambda_{a}\lambda_{b}\right)=\pm 2\;\delta_{ab}.$$

so that in a gauge theory of the simple superalgebra SU(*m/n*), either the SU(*m*) or the SU(*n*) matrices have a negative norm. Hence there is a risk of constructing states with a negative energy. But using $\tilde{D}=\chi (d+A)$, the sign of the time derivative in the Dirac equation is switched when we flip the sign of the chirality, maintaining the sign of the product energy x time. This restores the symmetry between SU(*m*) and SU(*n*). We conclude that both sectors, SU(*m*) and SU(*n*) follow the usual axioms and respect unitarity.

Note that in his many presentations, for example [9, 11], Alain Connes emphasizes the importance of the chirality in the definition of his Dirac-Yukawa operator. But although his analysis is closely related to the chiral differential $\tilde{d}=\chi d$ defined in equation (5.1), Connes in his works concerning the standard model of the fundamental interactions does not use simple superalgebras and does not make the connection to the SU(2/1) of Ne’eman [15] and Fairlie [16]. Maybe, our new formalism will help.

## 7 Chern-Simons Lagrangian

The standard construction of the Chern-Simons Lagrangian can be generalized to the chiral super connection. Consider the exterior form

(7.1) $$\tilde{C}=\tilde{A}\tilde{d}\tilde{A}+\frac{2}{3}\tilde{A}\tilde{A}\tilde{A}.$$

By taking the exterior differential we recover the usual formula

(7.2) $$STr(\tilde{d}\tilde{C})=STr(\tilde{F}\tilde{F})=g_{ab}{\overset{˘}{F}}^{a}{\overset{˘}{F}}^{b}+g_{ij}D{\text{Φ}}^{i}D{\text{Φ}}^{j},$$

where (*g*_*ab*_, *g*_*ij*_) is the super-Killing metric of the superalgebra (2.5) and $\overset{˘}{F}$ is defined in (5.3). Observe the natural occurrence of the supertrace, needed to recover the super-Killing metric. As usual, the term in *A*^4^ present in ${\tilde{F}}^{2}$ but not in $d\tilde{C}$ is eliminated by the Jacobi identity when we sum over the cyclic permutations of the indices (*def*)

(7.3) $${\text{Σ}}_{\text{def}}\;g_{ab}\;f_{cd}^{a}\;f_{ef}^{b}={\text{Σ}}_{\text{def}}\;f_{cda}\;f_{ef}^{a}=0.$$

In ${\overset{˘}{F}}^{2}$, we also have a term in ${\text{Φ}}^{4}$ which is eliminated by the super-Jacobi identity

(7.4) $${\text{Σ}}_{jkl}\;g_{ab}\;d_{ij}^{a}\;d_{kl}^{b}={\text{Σ}}_{jkl}\;f_{ija}\;d_{kl}^{a}=0.$$

The topological Lagrangian $STr(\tilde{F}\tilde{F})$ is as usual closed and locally exact. The unusual feature is that it is not a homogeneous 4-form but as in [2, 3] a differential exterior form of mixed degree, which interferes with the normal way in which we compute the Chern classes.

## 8 Discussion

Despite the importance of intrinsic differential geometry, and despite the abundance of textbooks and research devoted to superconnections (see for example [5, 6, 24]), we show in this paper that the construction of a connection valued in a Lie-Kac superalgebra [1] defined in terms of ordinary matrices, complex valued functions and ordinary differential forms can still be simplified. The method presented here differs from previous ones [2, 9] by a slight modification: in order to generate the correct signs in the definition of the curvature and hence recover the Bianchi identity as a consequence of the super-Jacobi identity, we have transferred in our definition $\tilde{D}=\chi (d+A)+\text{Φ}$ the commutation rule burden from the odd to the even generators. The odd field ${\text{Φ}}^{i}$ which carries the odd indices, is a normal commuting scalar field. However, the even field $\chi A_{\mu }^{a}$ carries a chirality operator χ which is an intrinsic element of any linear representation of a Lie-Kac superalgebra and which anticommutes with the odd generators (2.1). With this simple change, all the equations are explicitly covariant and are written in term of ordinary complex valued functions. This may open the way to new applications in geometry and quantum-field theory [22].
